# Interleukin-35 promotes Breg expansion and interleukin-10 production in CD19^+^ B cells in patients with ankylosing spondylitis

**DOI:** 10.1007/s10067-022-06137-8

**Published:** 2022-04-14

**Authors:** Yu Zhang, Sixi Wei, Qingqing Wu, Xue Shen, Wanrong Dai, Zhiqi Zhang, Man Chen, Hai Huang, Li Ma

**Affiliations:** 1 Center for Clinical Laboratories, The Affiliated Hospital of Guizhou Medical University, 28 Guiyi Street, Guiyang, 550004 Guizhou China; 2 Department of Clinical Biochemistry, School of Clinical Laboratory Science, Guizhou Medical University, 9 Beijing Road, Guiyang, 550004 Guizhou China; 3 Department of Microbiology and Immunology, School of Clinical Laboratory Science, Guizhou Medical University, 9 Beijing Road, Guiyang, 550004 Guizhou China; 4 Hebei Yanda Ludaopei Hospital, Sanhe, 065200 Hebei China

**Keywords:** Ankylosing spondylitis, Interleukin-10, Interleukin-35, Regulatory B cells

## Abstract

**Objective:**

IL-35 is a potent immunosuppressive and anti-inflammatory cytokine, consisting of a p35 subunit and an Epstein–Barr virus–induced gene 3 (EBI3) subunit, which suppresses CD4^+^ effector T cell proliferation and promotes regulatory T cell (Treg) expansion. However, the effects of IL-35 on regulatory B cells (Bregs) in ankylosing spondylitis (AS) have not been explored. The present study aimed (i) to measure serum IL-35 levels and the percentages of Bregs in the peripheral blood of patients with AS and (ii) to explore their relationships in the pathogenesis of AS.

**Methods:**

A total of 77 patients with AS (AS group), including 47 inactive AS and 30 active AS cases, and 59 healthy controls (HCs) were enrolled into this study. The serum levels of IL-35 and IL-10 were detected by ELISA, and the mRNA levels of p35 and EBI3 were measured by RT–qPCR. The percentages of CD19^+^CD24^hi^CD38^hi^ and CD19^+^CD24^hi^CD27^+^ Bregs and IL-35 receptor (IL-12Rβ2, IL-27Rα and gp130), IL-10, p-STAT1, p-STAT3, and p-STAT4 in CD19^+^ B cells were detected by flow cytometry. The correlations between IL-35 levels and percentages of Bregs were analyzed by determining Pearson’s correlation coefficient. The effect of IL-35 on Bregs was determined by mix-culture of recombinant (r) IL-35 with peripheral blood mononuclear cells (PBMCs).

**Results:**

The serum IL-35 and IL-10 levels, *p35* and *EBI3* mRNA levels, and the percentages of CD19^+^CD24^hi^CD38^hi^ and CD19^+^CD24^hi^CD27^+^ Bregs were significantly lower in AS patients than those in HCs. In addition, the percentages of CD19^+^CD24^hi^CD38^hi^ and CD19^+^CD24^hi^CD27^+^ Bregs in active AS patients were significantly lower than those in inactive AS patients. The serum IL-35 levels were positively correlated with the percentages of CD19^+^CD24^hi^CD38^hi^ and CD19^+^CD24^hi^CD27^+^ Bregs in AS patients. IL-12Rβ2 and IL-27Rα, but not gp130 subunit, were expressed in CD19^+^ B cells in AS patients. RIL-35 could effectively promote CD19^+^CD24^hi^CD38^hi^ Breg expansion and IL-10 production. Meanwhile, rIL-35 also promoted the expression of IL-12Rβ2 and IL-27Rα and the phosphorylation of STAT1 and STAT3 in CD19^+^ B cells.

**Conclusion:**

These results demonstrated that reduced IL-35 production may be associated with Bregs defects in AS patients. RIL-35 induced the proliferation of CD19^+^CD24^hi^CD38^hi^ Bregs and IL-10 production, suggesting that IL-35 may serve as a reference for further investigation to develop novel treatments for AS.

**Table:** 

**Key Points** • *Our study investigated the effects of IL-35 on Bregs in AS patients.* • *We found the serum IL-35, IL-10 levels, and the percentages of CD19*^+^*CD24*^*hi*^*CD38*^*hi*^* and CD19*^+^*CD24*^*hi*^*CD27*^+^ *Bregs were significantly lower in AS patients.* • *The serum IL-35 levels were positively correlated with the percentages of CD19*^+^*CD24*^*hi*^*CD38*^*hi*^* and CD19*^+^*CD24*^*hi*^*CD27*^+^ *Bregs in AS patients.* • *Recombinant IL-35 could effectively promote CD19*^+^*CD24*^*hi*^*CD38*^*hi*^* Breg expansion and IL-10 production.*

## Introduction

Ankylosing spondylitis (AS) is a common chronic inflammatory autoimmune disease that primarily affects the sacroiliac joints, spine and peripheral joints, and attachments of ligaments [1]. The main clinical characteristics are inflammation of the sacroiliac joints and spine, peripheral arthritis, and enthesitis, which may induce new bone formation and fusion of the affected area [2]. Over time, certain patients develop spinal immobility and ankylosis, which seriously affect patients’ quality of life. Currently, the precise etiopathogenesis of AS is not fully understood. Several lines of evidence suggest that susceptibility genes, environmental factors and loss of immune tolerance, and dysregulation of multiple cytokines are the principal determinants for its development. In particular, dysfunctional immune responses are crucial for the pathogenesis of AS [3, 4].

Regulatory B cells (Bregs), a unique subset of CD19^+^ B cells, have been revealed to possess immunosuppressive functions and support immunological tolerance. Bregs are able to secrete interleukin (IL)-10 and express molecules that inhibit the proliferation of pathogenic T cells, autoreactive B cells, and other proinflammatory lymphocytes, thus potently suppressing immunopathogenesis [5]. Recently, two major Breg subsets, i.e., CD19^+^CD24^hi^CD38^hi^ transitional B cells and CD19^+^CD24^hi^CD27^+^ memory B cells, both of which exhibit a regulatory capacity by expressing IL-10 upon stimulation ex vivo, were identified in humans [6–8]. Deficiency of Bregs may lead to multiple autoimmune diseases. Indeed, decreased Breg numbers and Breg dysfunction have been identified in juvenile idiopathic arthritis, Henoch–Schönlein purpura, Hashimoto’s thyroiditis (HT), and systemic sclerosis [9–12]. In addition, Xue et al. [13] found that the percentages of CD19^+^CD24^hi^CD38^hi^ Bregs in peripheral blood of AS patients were reduced compared to those of healthy controls (HCs). Chen et al. [14] recently reported a functional defect of CD19^+^CD24^+^CD38^+^ Bregs in patients with AS, which impaired their capacity to produce IL-10. These findings suggest that a decrease or a functional defect of Bregs may participate in the pathogenesis of AS.

IL-35, a heterodimeric cytokine composed of p35 and Epstein–Barr virus–induced gene 3 (EBI3) subunits, was recently identified as a new member of the IL-12 cytokine family, which also comprises IL-12, IL-23, and IL-27 [15]. It has anti-inflammatory and immunosuppressive properties and plays an important role in the development and prevention of various infectious and autoimmune diseases [16, 17]. IL-35 exerts an anti-inflammatory effect by inhibiting CD4^+^ effector T cell (Teff) proliferation, modulating T cell differentiation, and inducing regulatory T cells (Tregs). Moreover, in mice with experimental autoimmune uveitis, IL-35 has been revealed to induce IL-10 secretion in B cells and expand the number of Bregs [18]. Recombinant (r) IL-35 induces Bregs to produce IL-10 [19]. IL-35 is also critical to Breg-mediated protection against autoimmune diseases.

The expression and regulation of IL-35 are regulated by its ligand and its downstream signaling pathway. IL-35 has been shown to mediate intracellular signaling through either the heterodimer of receptor chains IL-12Rβ2:gp130 or homodimers of each chain (IL-12Rβ2:IL-12Rβ2, gp130:gp130), and IL-35 receptor (IL-35R) signaling is mainly mediated by pathways involving the transcription factors signal transducer and activator of transcription 1 (STAT1) and STAT4 or STAT1 or STAT4 separately [20, 21]. However, Ma et al. [22] found that IL-35 in T cells was capable of activating phosphorylation of both STAT1 and STAT3. A further study found that the binding of IL-35 to the IL-12Rβ2:IL-27Rα (WSX-1) heterodimer can induce the activation of STAT1 and STAT3, but not STAT4, in B cells [18].

Additional evidence indicated that decreased IL-35 levels may contribute to the development of autoimmune disease. However, the role of IL-35 in the occurrence and development of different diseases is not yet clear. The effects of IL-35 on Bregs in AS have remained unexplored. In this study, we first analyzed the levels of serum IL-35 and the percentages of peripheral blood Breg subsets in AS patients with different active disease. In addition, we evaluated the correlations between the serum levels of IL-35 and the percentages of Breg subsets and further examined the effects of IL-35 on Bregs proliferation and IL-10 production in patients with AS. The present findings provide useful insights in the role of IL-35 in AS and may serve as a reference for further investigation to develop novel treatments for AS.

## Materials and methods

### Patients and healthy controls

From September 2017 to March 2019, a total of 77 untreated AS patients and 59 matched HCs were enrolled in the current study. The diagnosis of AS was established following the 1984 American College of Rheumatology classification criteria [23]. All subjects with infections, tumors, and other autoimmune or rheumatologic diseases were excluded. Moreover, patients who had received prior treatment with non-steroidal anti-inflammatory drugs, steroids, or other immunosuppressants were excluded. The Bath AS Disease Activity Index (BASDAI) scoring system was used to evaluate the disease activity status [24]. The scores for each criterion ranged from 0 to 10. Active AS was defined as BASDAI score ≥ 4 and inactive AS as BASDAI score < 4. Of the 77 AS patients, 30 were classified as active AS and 47 as inactive AS patients. By adopting the questionnaires, epidemiological information of AS patients was obtained. Disease duration was defined as the interval from the time of symptom appearance to this study. The laboratory parameters, including erythrocyte sedimentation rate (ESR) and hypersensitive C-reactive protein (Hs-CRP) levels, and other clinical parameters were collected at the same time. The clinical characteristics of the AS patients and HCs are shown in Table 1. The experimental group and the control group showed no significant differences with respect to gender and age. The study was approved by the Ethical Committee of The Affiliated Hospital of Guizhou Medical University (Guiyang, China) and was performed in accordance with the Declaration of Helsinki. Written informed consent was obtained from all patients and HCs for the publication of the current study.

**Table 1:** Clinical characteristics of AS patients and healthy controls

Variable	HCs (*n* = 59)	Inactive AS (*n* = 47)	Active AS (*n* = 30)	F/χ^2^/t	*P*
Age (years)	27.40 ± 5.01	27.30 ± 8.22	30.66 ± 8.03	2.639	0.075
Males/females	43/16	42/5	25/5	4.745	0.093
Duration of disease (years)	NA	4.33 ± 4.48	7.65 ± 7.03^*^	2.537	0.013
ESR (mm/h)	NA	14.94 ± 8.48	33.67 ± 12.19^**^	7.354	< 0.001
Hs-CRP (mg/l)	NA	4.78 ± 4.93	20.84 ± 12.33^**^	8.003	< 0.001
BASDAI score (0–10)	NA	2.44 ± 0.86	5.12 ± 0.87^**^	13.270	< 0.001

*HC* healthy control, *AS* ankylosing spondylitis, *NA* not available, *ESR* erythrocyte sedimentation rate, *Hs-CRP* hypersensitive C-reactive protein, *BASDAI* Bath Ankylosing Spondylitis Disease Activity Index

^*^P < 0.05, ^**^P < 0.01 vs. the inactive AS group

### Sample collection

Fasting venous blood samples were collected from each participant between 8:00 and 9:00 a.m. One part of each blood sample was collected in non-anticoagulant tubes (5 ml), centrifuged (3500 × *g* for 10 min at room temperature) to collect serum specimens, and stored at − 80 °C until further analysis. Another part was collected in Vacutainer tubes (5 ml) containing ethylenediamine tetra-acetic acid (EDTA) for flow cytometry analysis, and the remainder was used to prepare peripheral blood mononuclear cells (PBMCs). All blood samples were processed within 4 h after collection.

### ELISA detection of cytokines

The serum IL-35 and IL-10 levels were measured using specific ELISA kits (Elabscience, Wuhan, China) following the manufacturer’s instructions. Each sample was tested in triplicate. The optical density was measured at 450 nm using an automatic ELISA reader (Bio-Rad Laboratories, Inc.). A standard curve was generated for each plate, and the absolute concentrations of IL-35 and IL-10 were calculated. The results were expressed as picograms per milliliter.

### RT–qPCR

Total mRNA was isolated from PBMCs with TRIzol reagent (Invitrogen; Thermo Fisher Scientific, Inc.) in accordance with the manufacturer’s protocol. The concentration and purity of mRNA were measured by reading the absorbance at 260 nm and 280 nm. mRNA samples with A_260_/A_280_ ratios between 1.8 and 2.0 were then reverse transcribed using a PrimeScript RT Reagent Kit (Takara Biotechnology Co., Ltd.) following the manufacturer’s protocol. The cDNA samples were stored at − 80 °C until further analysis. Reverse transcription–quantitative PCR (RT–qPCR) was performed on an Applied Biosystems 7500 Real-Time PCR system (Applied Biosystems; Thermo Fisher Scientific, Inc.) using SYBR® Premix Ex Taq™ II (Takara Biotechnology Co., Ltd.), according to the manufacturer’s guidelines. The primer sequences of *p35*, *EBI3*, and *β-actin* (reference gene) (Shanghai Shenggong Biology Co., Ltd.) were as follows: *EBI3* forward 5ʹ-CACTGAAGTACTGGATCCGT-3ʹ and reverse 5ʹ-GGAGACTCCAGTCACTCAGT-3ʹ; *p35* forward 5ʹ-CAGGTGGAGTTCAAGACCAT-3ʹ and reverse 5ʹ-CCGGTTCTTCAAGGGAGGAT-3ʹ; and *β-actin* forward 5ʹ-TAGTTGCGTTACACCCTTTCTTG-3ʹ and reverse 5ʹ-TCACCTTCACCGTTCCAGTTT-3ʹ. The thermocycling steps were as follows: denaturation at 95 °C for 30 s, followed by 40 cycles at 95 °C for 5 s and 60 °C for 30 s. The amplification and melting curves were checked after the reaction. All experiments were done in triplicate. Gene expression was normalized to *β-actin* and relative expression levels were evaluated using the 2^−ΔΔCt^ method.

### Cell cultures and stimulation assays

PBMCs were isolated by standard Ficoll density gradient centrifugation from blood samples from 35 untreated AS patients. Freshly isolated PBMCs were incubated in RPMI 1640 medium (HyClone; GE Healthcare Life Sciences) supplemented with 10% fetal bovine serum (FBS) (Gibco; Thermo Fisher Scientific, Inc.) and penicillin/streptomycin (Solarbio; Beijing Solarbio Science & Technology Co., Ltd.). PBMCs were seeded into 24-well plates (1 × 10^6^ cells/ml/well). Each patient’s PBMCs were divided into two groups and cultured with (i) phosphate-buffered saline (PBS) (control group) or (ii) rIL-35 (100 ng/ml) (Sino Biological, Inc.) (experimental group) at 37 °C, 5% CO_2_ for 48 h. Cell culture supernatants were collected after 48 h. Supernatant samples were stored at − 80 °C until later batched analysis. Cells were analyzed by flow cytometry.

### Flow cytometry analysis for CD19^+^CD24^hi^CD38^hi^ Bregs, CD19^+^CD24^hi^CD27^+^ Bregs, and IL-35R

Peripheral venous blood was collected using EDTA as an anticoagulant. The following antibodies were used for surface staining: fluorescein isothiocyanate (FITC)–conjugated anti-CD27 (cat. no. 340424; BD Bioscience), phycoerythrin (PE)–conjugated anti-CD24 (cat. no. 555428; BD Pharmingen; BD Biosciences), peridinin–chlorophyll–protein complex (PerCP)–cyanine (cy)5.5–conjugated anti-CD45 (cat. no. 652803; BD Biosciences), allophycocyanin (APC)–conjugated anti-CD38 (cat. no. 345807; BD Biosciences), PerCP–cy5.5–conjugated anti-IL-12Rβ2 (cat. no. FAB1959C; R&D Systems, Inc.), APC-conjugated anti-gp130 (cat. no. FAB228A; R&D Systems, Inc.), PE-conjugated anti-IL-27Rα (cat. no. FAB14791P; R&D Systems, Inc.), APC-cy7-conjugated anti-CD4 (cat. no. 341115; BD Biosciences), and PE–cy7–conjugated anti-CD19 (cat. no. 341113; BD Biosciences). Then, the cells were incubated with the above indicated surface antibodies for 15 min at room temperature in the dark. Control staining was performed with the following isotype-matched control antibodies: PerCP–cy5.5–conjugated anti-immunoglobulin G (IgG)1 (cat. no. 347202; BD Biosciences), FITC-conjugated anti-IgG (cat. no. 349041; BD Biosciences), and PE-conjugated anti-IgG1 (cat. no. 349043; BD Biosciences), APC-conjugated anti-IgG (cat. no. 555751; BD Bioscience). Controls were isotyped, which helped eliminate nonspecific fluorescent interference in flow analysis, and an isotype-matched negative control was used for each sample. In addition, in order to lyse erythrocytes, the stained samples were treated with FACS™ lysing solution (cat. no. 349202; BD Biosciences) for 7 min at room temperature in the dark. Cells were washed with PBS twice and then analyzed by flow cytometry using a FACS Canto II instrument (BD Biosciences), and data were analyzed by FACS Diva software (version 6.1.3; BD Biosciences).

### Flow cytometry analysis for the phosphorylated signal transducer and activator of transcription 1 (p-STAT1), p-STAT3, p-STAT4, and IL-10 protein levels in CD19^+^ B cells

PBMCs (1 × 10^6^ cells/ml) were stimulated for 48 h with rIL-35 (100 ng/ml; Sino Biological, Inc.). The cultured cells in duplicate were stained with surface FITC-conjugated anti-CD3 (cat. no. 349201; BD Biosciences) and PE–cy7–conjugated anti-CD19 (cat. no. 341113; BD Biosciences) at room temperature for 15 min in the dark. The cells were subsequently fixed with 50 µl BD FACS™ permeabilizing solution A (cat. no. 347692; BD Biosciences) at room temperature for 5 min in the dark according to the manufacturer’s instructions and then permeabilized with BD FACS™ permeabilizing solution B (cat. no. 347692; BD Biosciences) and incubated with intracellular APC-conjugated anti-IL-10 (cat. no. 554707; BD Pharmingen; BD Biosciences), PE-conjugated anti-phospho-STAT1 (anti-p-STAT) (cat. no. 8062; Cell Signaling Technology, Inc.), PE-conjugated anti-p-STAT3 (cat. no. 87544; Cell Signaling Technology, Inc.), PE-conjugated anti-p-STAT4 (cat. no. 13223; Cell Signaling Technology, Inc.), or isotype-matched controls at room temperature for 30 min in the dark. After washing with PBS, the cells were subjected to flow cytometry analysis using a FACS Canto II instrument (BD Biosciences), and data were analyzed by FACS Diva software (version 6.1.3; BD Biosciences).

### Analysis of the effect of IL-35 on the percentages of Breg subsets and IL-10 levels

To investigate the effect of IL-35 on the percentages of CD19^+^CD24^hi^CD38^hi^ Bregs, CD19^+^CD24^hi^CD27^+^ Bregs, and IL-10 levels in vitro, PBMCs (1 × 10^6^ cells/ml) were stimulated for 48 h with rIL-35 (100 ng/ml; Sino Biological, Inc.). Then, the stimulated cells and supernatant samples were collected. The percentages of CD19^+^CD24^hi^CD38^hi^ Bregs and CD19^+^CD24^hi^CD27^+^ Bregs were analyzed using flow cytometry, and supernatant IL-10 levels were measured using a specific ELISA. The method of operation is as described above.

### Statistical analysis

All statistical analyses were performed using SPSS version 24.0 (SPSS, Inc., Chicago, IL, USA) and GraphPad Prism version 7.0 software (GraphPad Software, Inc., La Jolla, CA, USA). A Kolmogorov–Smirnov test of normality was performed for all variables. Results are expressed as mean standard deviation for normally distributed data. Numerical data are expressed as median (interquartile range) if they were not normally distributed. Comparisons between groups were performed using the unpaired Student *t*-test or the Mann–Whitney *U* test. Pearson’s correlation and Spearman’s rank correlation were used in cases of normally and non-normally distributed data, respectively. A two-sided *P* < 0.05 was considered statistically significant.

## Results

### Clinical characteristics of subjects

The clinical characteristics of AS patients and HCs are shown in Table 1. The 77 untreated AS patients were first stratified into two subgroups (inactive AS or active AS) based on disease activity as reflected by the BASDAI scores. Patients with active AS had a longer disease duration compared to patients with inactive AS (*t* = 2.537, *P* = 0.013). In addition, the ESR and Hs-CRP levels were higher in the active AS group than in the inactive AS group (*t*1 = 7.354, *P*1 < 0.001; *t*2 = 8.003, *P*2 < 0.001). There was no significant difference in age and gender ratio between inactive/active AS patients and HCs (*P* > 0.05).

### Serum IL-35 and IL-10 levels were lower in patients with AS

**Fig. 1:**
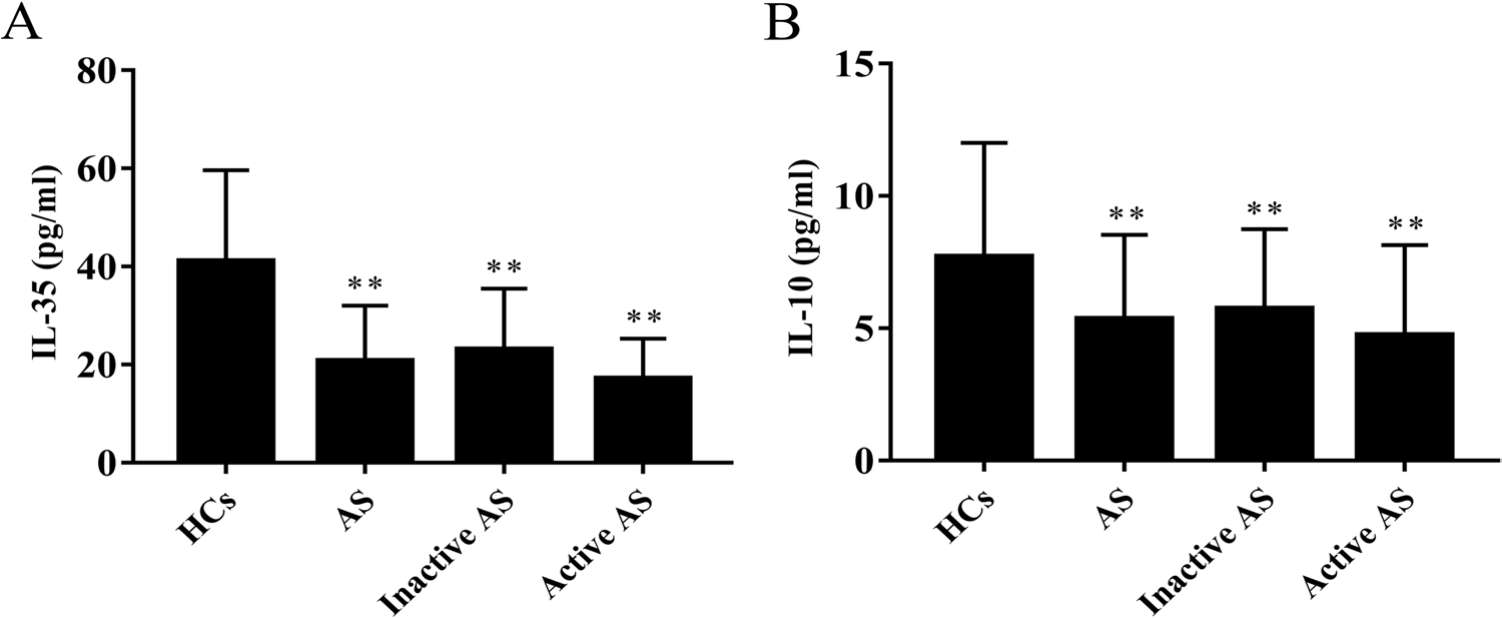
Serum IL-35 and IL-10 levels in AS patients (*n* = 77) and HCs (*n* = 59). Patients with AS were further divided into inactive AS (*n* = 47) and active AS (*n* = 30). IL-35 and IL-10 were detected by ELISA. The levels of **A** IL-35 and **B** IL-10 were compared between AS patients and HCs. ^*^P < 0.05, ^**^P < 0.01 vs. the HC group. ^#^*P* < 0.05, ^##^*P* < 0.01 vs. the inactive AS group

We first examined the serum levels of IL-35 in untreated AS patients. They were significantly lower in patients with inactive/active AS than in HCs (*t*1 = 5.703, *P*1 < 0.001; *t*2 = 6.727, *P*2 < 0.001) (Fig. 1A). There was no significant difference between patients with inactive AS and patients with active AS (*P* > 0.05). Serum IL-10 levels were also significantly lower in inactive/active AS patients compared with HCs (*t*1 = 2.660, *P*1 = 0.009; *t*2 = 3.553, *P*2 = 0.001) (Fig. 1B). There was no significant difference in IL-10 serum levels between inactive and active AS patients (*P* > 0.05).

### mRNA levels of p35 and EBI3 were lower in patients with AS

**Fig. 2:**
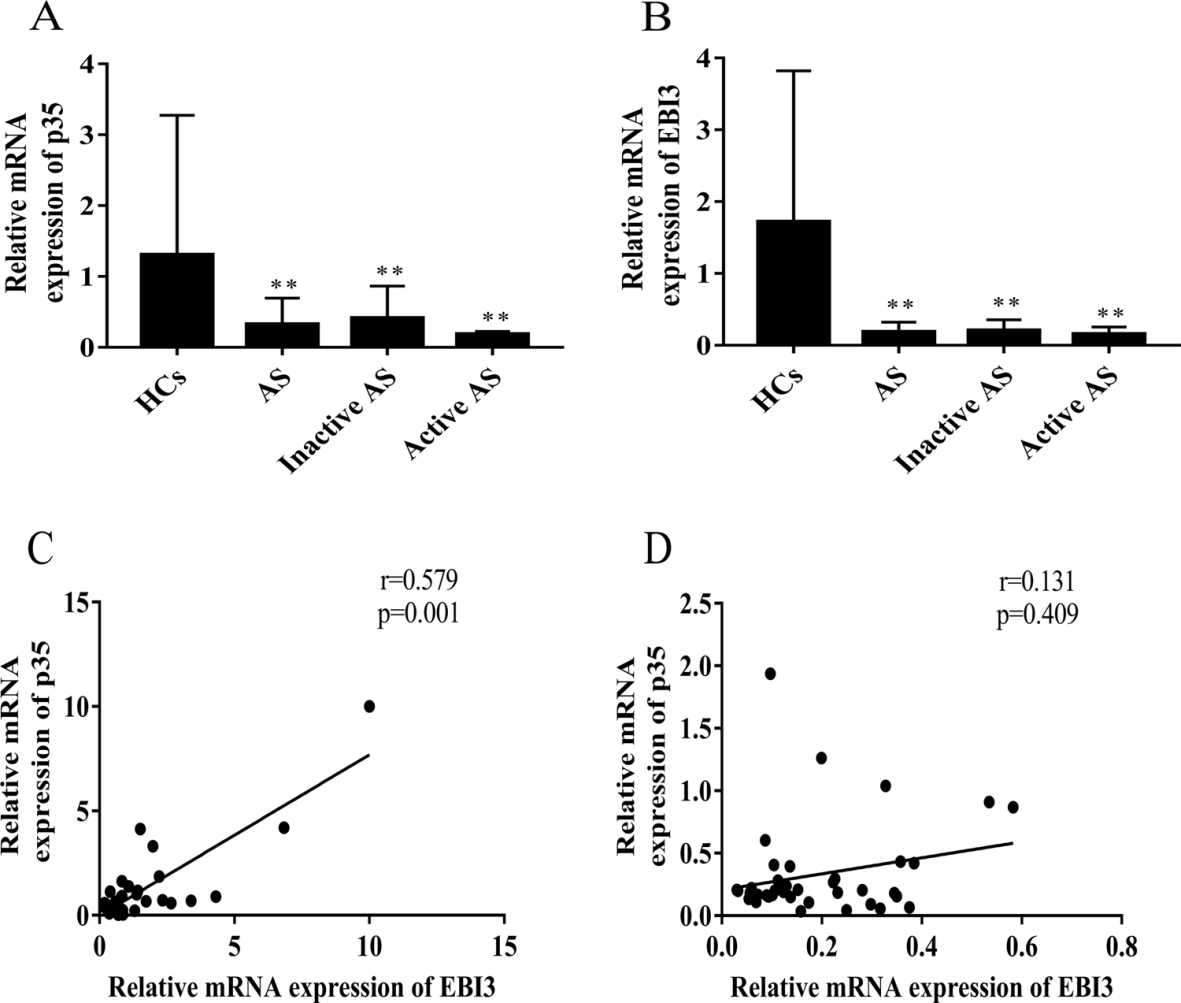
The relative mRNA levels of *p35* and *EBI3* in isolated PBMCs from AS patients (*n* = 42) and HCs (*n* = 30). Patients with AS were further divided into inactive AS (*n* = 26) and active AS (*n* = 16) groups. Relative mRNA expression of *p35* and *EBI3* was determined by reverse transcription–quantitative PCR and normalization to *β-actin*. The mRNA levels of **A** *p35* and **B** *EBI3* were compared between AS patients and HCs. **C** and **D** Correlations between *p35* and *EBI3* mRNA in **C** HCs and **D** patients with AS. ^*^P < 0.05, ^**^P < 0.01 vs. the HC group. ^#^*P* < 0.05, ^##^*P* < 0.01 vs. the inactive AS group

IL-35 is composed of p35 and EBI3 subunits. The mRNA levels of *p35* and *EBI3* were assayed in PBMCs from AS patients and HCs and normalized to *β-actin*. The mRNA levels of *p35* and *EBI3* were lower in inactive/active AS patients than those in HCs (*Z*1 =  − 5.750, *P*1 < 0.001; *Z*2 =  − 5.189, *P2* < 0.001; *Z*3 =  − 2.793, *P*3 = 0.005; *Z*4 =  − 3.667, *P*4 < 0.001) (Fig. 2A and 2B). However, no significant difference was observed in the mRNA levels of *p35* and *EBI3* between the inactive and active AS groups (*P* > 0.05). A positive correlation was observed between *p35* and *EBI3* mRNA levels in HCs (*r* = 0.579, *P* = 0.001) (Fig. 2C). However, there was no significant correlation between *p35* and *EBI3* mRNA levels in patients with AS (*r* = 0.131, *P* = 0.409) (Fig. 2D). These results implied that the downregulation of *p35* and *EBI3* mRNA is closely related to the decrease of IL-35 levels in AS patients.

### Percentages of Breg subsets were lower in patients with AS

**Fig. 3:**
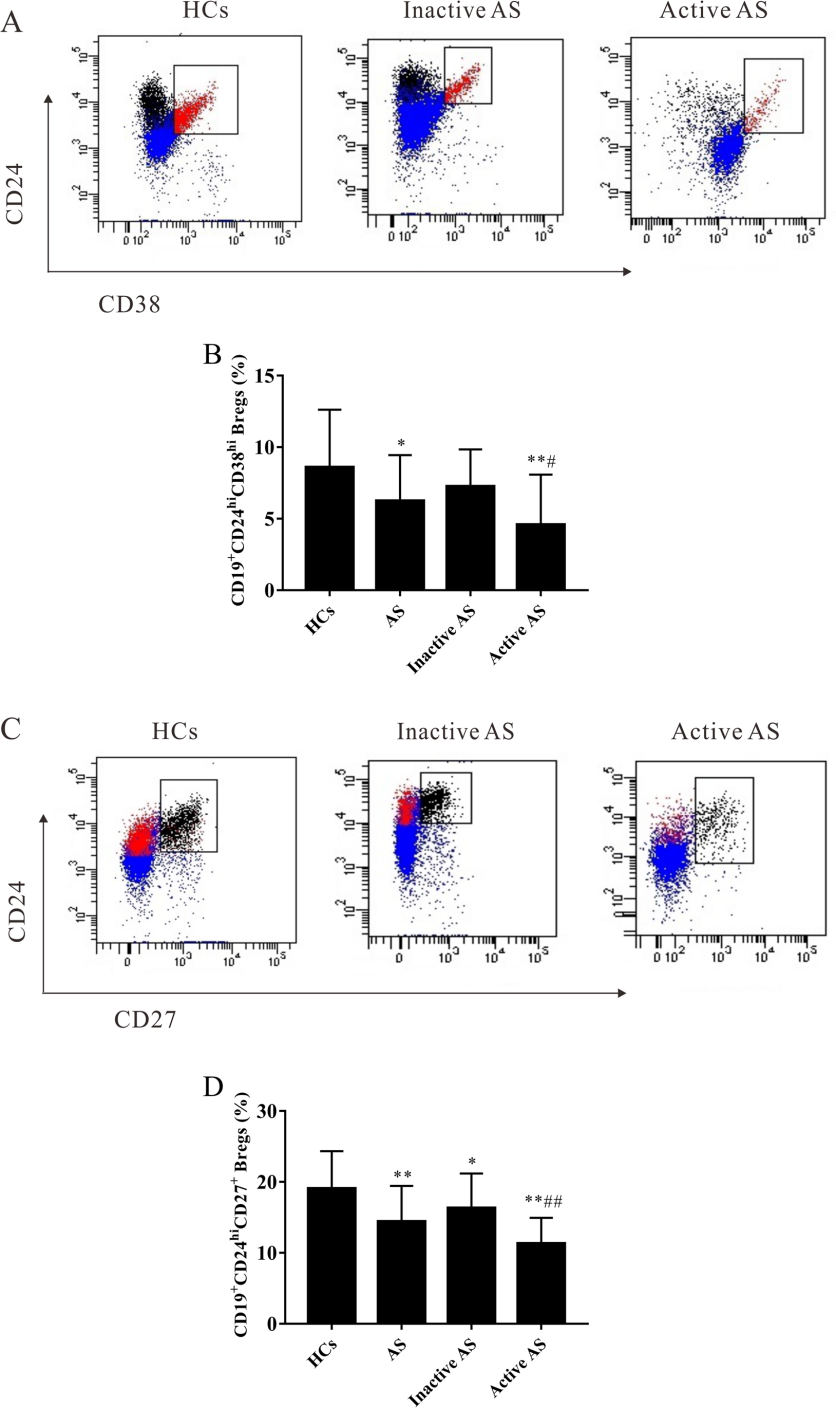
The abundance of CD19^+^CD24^hi^CD38^hi^ Bregs, and CD19^+^CD24^hi^CD27^+^ Bregs in patients with AS (*n* = 42) and HCs (*n* = 30). Patients with AS were further divided into inactive AS (*n* = 26) and active AS (*n* = 16). Representative FACS images of **A** CD19^+^CD24^hi^CD38^hi^ Bregs, and **C** CD19^+^CD24^hi^CD27^+^ Bregs. Abundance of **B** CD19^+^CD24^hi^CD38^hi^ Bregs, and **D** CD19^+^CD24^hi^CD27^+^ Bregs was compared between AS patients and HCs. ^*^P < 0.05, ^**^P < 0.01 vs. the HC group. ^#^*P* < 0.05, ^##^*P* < 0.01 vs. the inactive AS group

Next, the percentages of peripheral blood Breg subsets in patients with AS were analyzed using flow cytometry (Fig. 3A and 3C). The results showed that the percentage of CD19^+^CD24^hi^CD38^hi^ Bregs in CD19^+^ B cells was significantly lower in patients with AS compared with HCs (*t* = 2.661, *P* = 0.010) (Fig. 3B), and the percentages of CD19^+^CD24^hi^CD38^hi^ Bregs in CD19^+^ B cells were significantly lower in patients with active AS compared with HCs and patients with inactive AS (*t*1 =  − 3.513, *P*1 = 0.001; *t*2 =  − 2.404, *P*2 = 0.023) (Fig. 3B). However, there was no significant difference observed in the percentages of CD19^+^CD24^hi^CD38^hi^ Bregs in CD19^+^ B cells between HCs and patients with inactive AS (*P* > 0.05) (Fig. 3B). In addition, the percentages of CD19^+^CD24^hi^CD27^+^ Bregs in CD19^+^ B cells were lower in patients with inactive/active AS compared with HCs (*t*1 =  − 2.018, *P*1 = 0.049; *t*2 =  − 5.846, *P*2 < 0.001) (Fig. 3D). Significantly lower percentages of CD19^+^CD24^hi^CD27^+^ Bregs in CD19^+^ B cells were also observed in patients with active AS compared to those in patients with inactive AS (*t* =  − 2.491, *P* = 0.19) (Fig. 3D).

### Correlation between IL-35 serum levels and the percentages of Breg subsets in AS patients

**Fig. 4:**
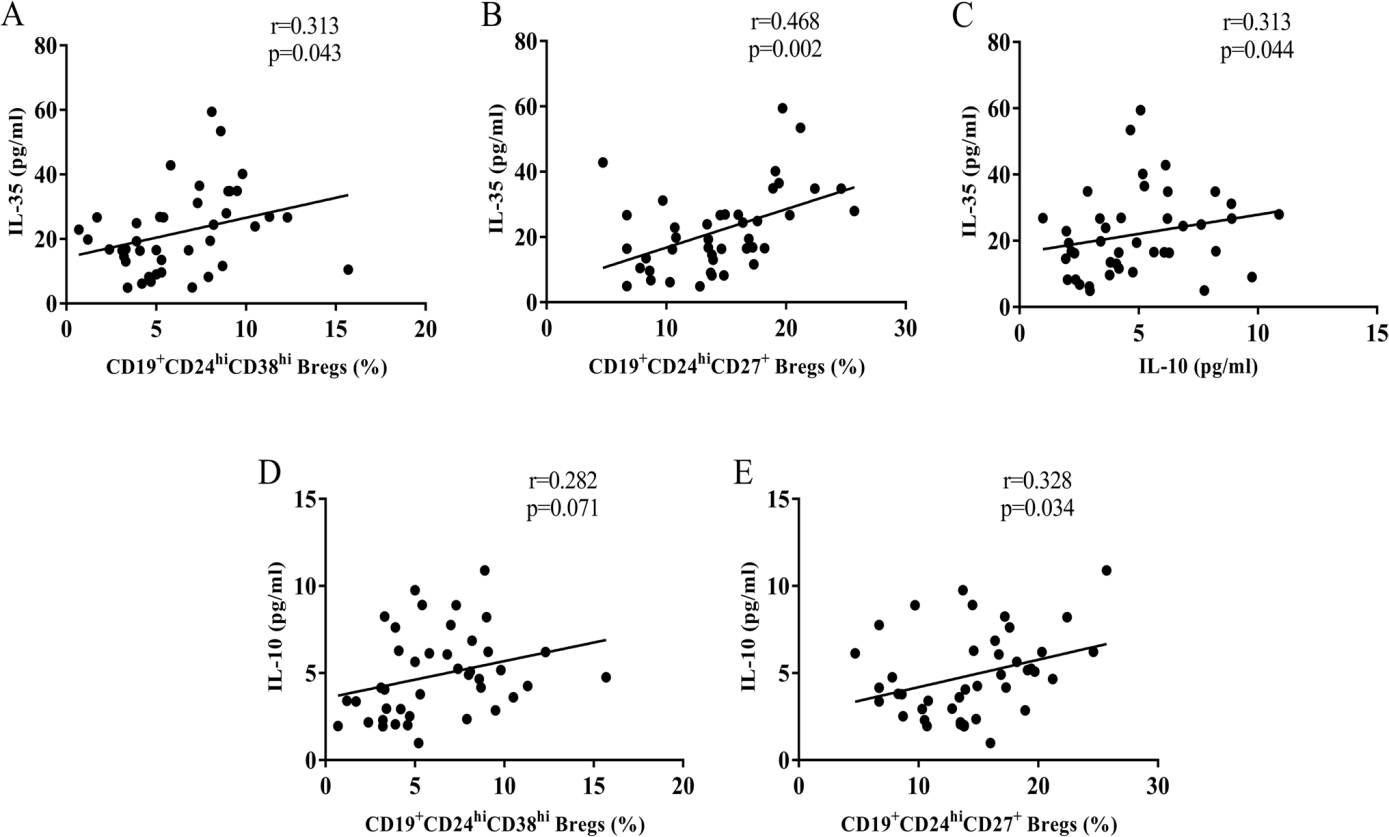
Correlation between (i) IL-35 serum levels and (ii) the percentages of CD19^+^CD24^hi^CD38^hi^ Bregs, CD19^+^CD24^hi^CD27^+^ Bregs, and IL-10 serum levels in patients with AS (*n* = 42). Pearson’s correlation coefficient was determined in the correlation analysis. The IL-35 serum levels were positively correlated with the percentages of **A** CD19^+^CD24^hi^CD38^hi^ Bregs (*r* = 0.313, *P* = 0.043) and **B** CD19^+^CD24^hi^CD27^+^ Bregs (*r* = 0.468, *P* = 0.002) and **C** the IL-10 serum levels (*r* = 0.313, *P* = 0.044). **D** There were no significant correlations between the percentages of CD19^+^CD24^hi^CD38^hi^ Bregs and IL-10 serum levels (*r* = 0.282, *P* = 0.071). **E **the percentages of CD19^+^CD24^hi^CD27^+^ Bregs were positively correlated with the IL-10 serum levels (*r* = 0.328, *P* = 0.034)

We examined the correlations between (i) IL-35 serum levels and (ii) IL-10 serum levels and the percentages of CD9^+^CD24^hi^CD38^hi^ and CD19^+^CD24^hi^CD27^+^ Bregs. Pearson correlation analysis revealed that the IL-35 serum levels were positively correlated with the percentages of CD19^+^CD24^hi^CD38^hi^ and CD19^+^CD24^hi^CD27^+^ Bregs and IL-10 serum levels (*r*1 = 0.313, *P*1 = 0.043; *r*2 = 0.468, *P*2 = 0.002; *r*3 = 0.313, *P*3 = 0.044) (Fig. 4A–C). These results indicated that decreases in the percentages of both CD19^+^CD24^hi^CD38^hi^ and CD19^+^CD24^hi^CD27^+^ Bregs may be associated with reduced IL-35 levels in patients with AS. Furthermore, the percentages of CD19^+^CD24^hi^CD27^+^ Bregs were positively correlated with the IL-10 serum levels (*r* = 0.328, *P* = 0.034) (Fig. 4E), and there were no significant correlations between CD19^+^CD24^hi^CD38^hi^ Breg counts and IL-10 serum levels (*r* = 0.282, *P* = 0.071) (Fig. 4D).

### Correlation between IL-35, IL-10, CD19^+^CD24^hi^CD38^hi^ Bregs, CD19^+^CD24^hi^CD27^+^ Bregs, and disease activity scores of BASDAI in AS patients

**Fig. 5:**
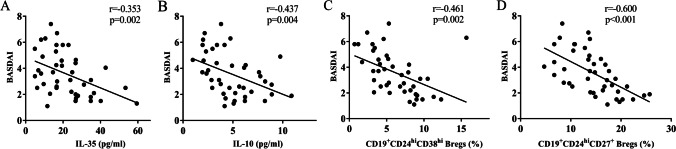
Correlation between IL-35, IL-10, CD19^+^CD24^hi^CD38^hi^ Bregs, CD19^+^CD24^hi^CD27^+^ Bregs, and disease activity in AS patients (*n* = 42). Pearson’s correlation coefficient was determined in the correlation analysis. **A** The IL-35 serum levels were negatively associated with the BASDAI score (*r* =  − 0.353, *P* = 0.002) in AS patients. **B** The IL-10 serum levels were negatively associated with the BASDAI score (*r* =  − 0.437, *P* = 0.004) in AS patients. **C** The percentage of CD19^+^CD24^hi^CD38^hi^ Bregs was negatively associated with the BASDAI score (*r* =  − 0.461, *P* = 0.002) in AS patients. **D** The percentage of CD19^+^CD24^hi^CD27^+^ Bregs was negatively associated with the BASDAI score (*r* =  − 0.461, *P* = 0.002) in AS patients

The serum levels of IL-35 and IL-10 were negatively associated with the BASDAI scores (*r* =  − 0.353, *P* = 0.002; *r* =  − 0.437, *P* = 0.004) (Fig. 5A and 5B). Similarly, the percentages of CD19^+^CD24^hi^CD38^hi^ and CD19^+^CD24^hi^CD27^+^ Bregs were also negatively associated with the BASDAI scores (*r* =  − 0.461, *P* = 0.002; *r* =  − 0.600, *P* < 0.001) (Fig. 5C and 5D).

### IL-35 induces expression of IL-10 in CD19^+^ B cells

**Fig. 6:**
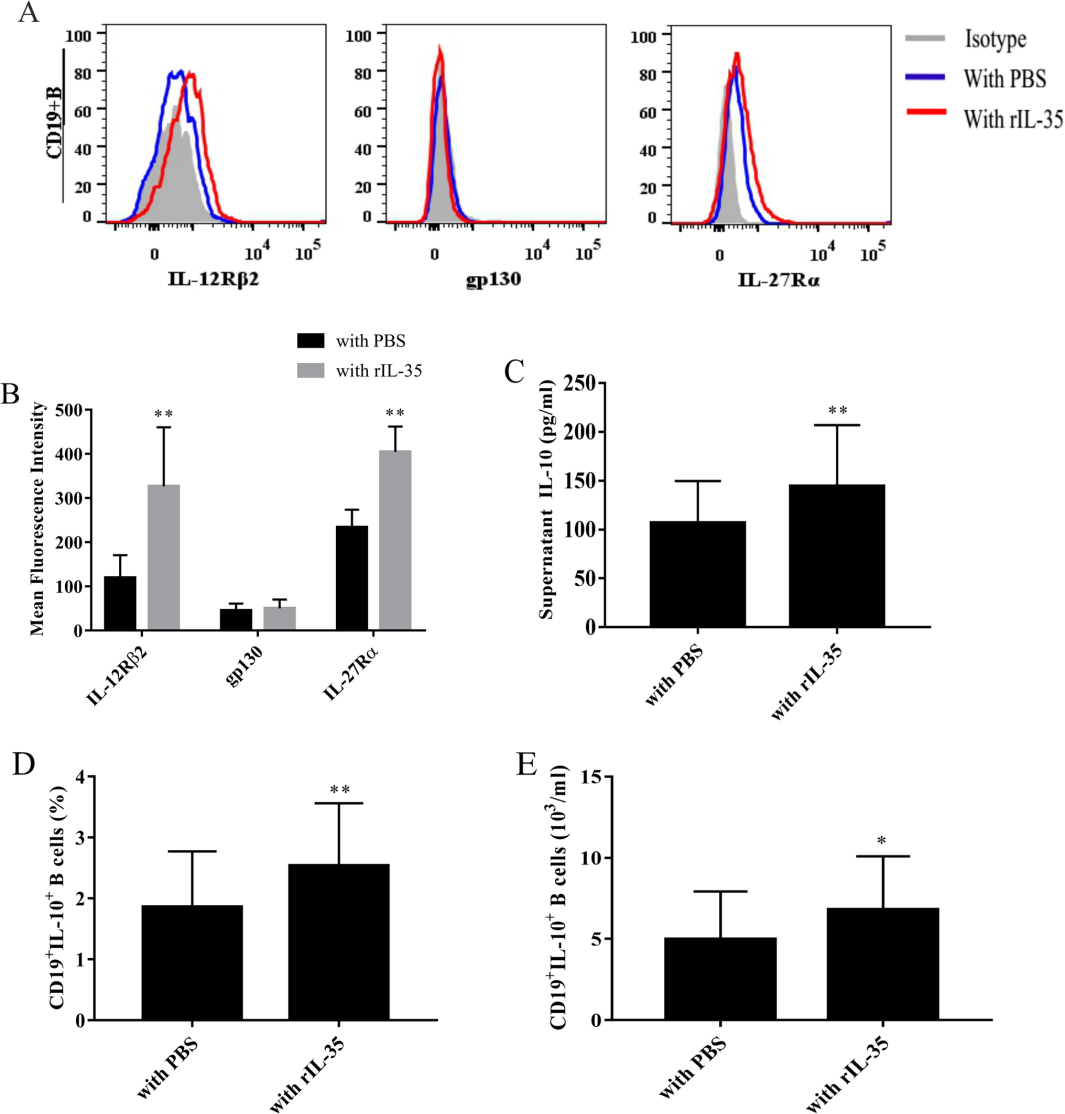
IL-35 induces expression of IL-10 in CD19^+^ B cells in PBMCs from patients with AS (*n* = 35). **A** The protein expression levels of IL-35R chains (IL-12Rβ2, gp130, and IL-27Rα) in CD19^+^ B cells were analyzed by flow cytometry. The IL-12Rβ2 and IL-27Rα chains, but not the gp130 chain, were present in CD19^+^ B cells from peripheral blood of AS patients. **B** The mean fluorescence intensity (MFI) of IL-12Rβ2 and IL-27Rα was significantly increased after rIL-35 stimulation compared to PBS treatment. **C** The IL-10 levels in supernatant and **D** the percentage of CD19^+^IL-10^+^ B cells were significantly higher in the rIL-35 group than those in the PBS group. ^*^P < 0.05, ^**^P < 0.01 compared with the PBS group

After showing that there is a significant positive correlation between IL-35 levels and the percentages of Breg subsets in AS patients, we next determined whether IL-35 contributed to the expansion of Bregs. We analyzed whether CD19^+^ B cells express receptors that interact with IL-35. Flow cytometry analysis revealed that IL-12Rβ2 and IL-27Rα chains, but not the gp130 chain, were present in all CD19^+^ B cells from peripheral blood of AS patients (Fig. 6A). Furthermore, the expression levels of IL-35 receptor subunits IL-12Rβ2 and IL-27Rα on the surface of CD19^+^ B cells from peripheral blood of AS patients were significantly upregulated in the rIL-35–stimulated group compared to the PBS-treated group (*Z*1 =  − 6.032, *P*1 < 0.001; *Z*2 =  − 7.007, *P*2 < 0.001) (Fig. 6B).

A previous report [22] demonstrated that rIL-35 effectively induced cytokine production in healthy PBMCs. In the present study, 10^6^ of purified PBMCs from 35 AS patients were stimulated with rIL-35 and PBS for 48 h. IL-10 levels in the cultured supernatants were measured by ELISA. We found that supernatant IL-10 levels were increased in rIL-35–stimulated PBMCs compared to PBS-treated PBMCs (*Z* =  − 2.437, *P* = 0.015) (Fig. 6C). The identification of Bregs is now dependent on their ability to produce IL-10. Interestingly, the percentages of CD9^+^IL-10^+^ B cells in CD19^+^ B cells from AS patients were significantly higher after rIL-35 stimulation compared to PBS treatment (*t* =  − 2.904, *P* = 0.005) (Fig. 6D) and the absolute number of CD9^+^IL-10^+^ B cells were also significantly higher in the rIL-35-stimulated group (*t* = −2.447, *P* = 0.017) (Fig. 6E), which indicated the elevation of IL-10 levels could be attributed to CD19^+^ B cells. The enhanced expression of IL-10–producing CD19^+^ B cells may be attributed to the increased percentages of Bregs cells induced by IL-35. These data suggest that the reduced IL-10 production in AS patients is either associated with a decrease in the number of Bregs or due to an intracellular functional defect.

### The percentage of CD19^+^CD24^hi^CD38^hi^ Bregs was increased by stimulation with rIL-35

**Fig. 7:**
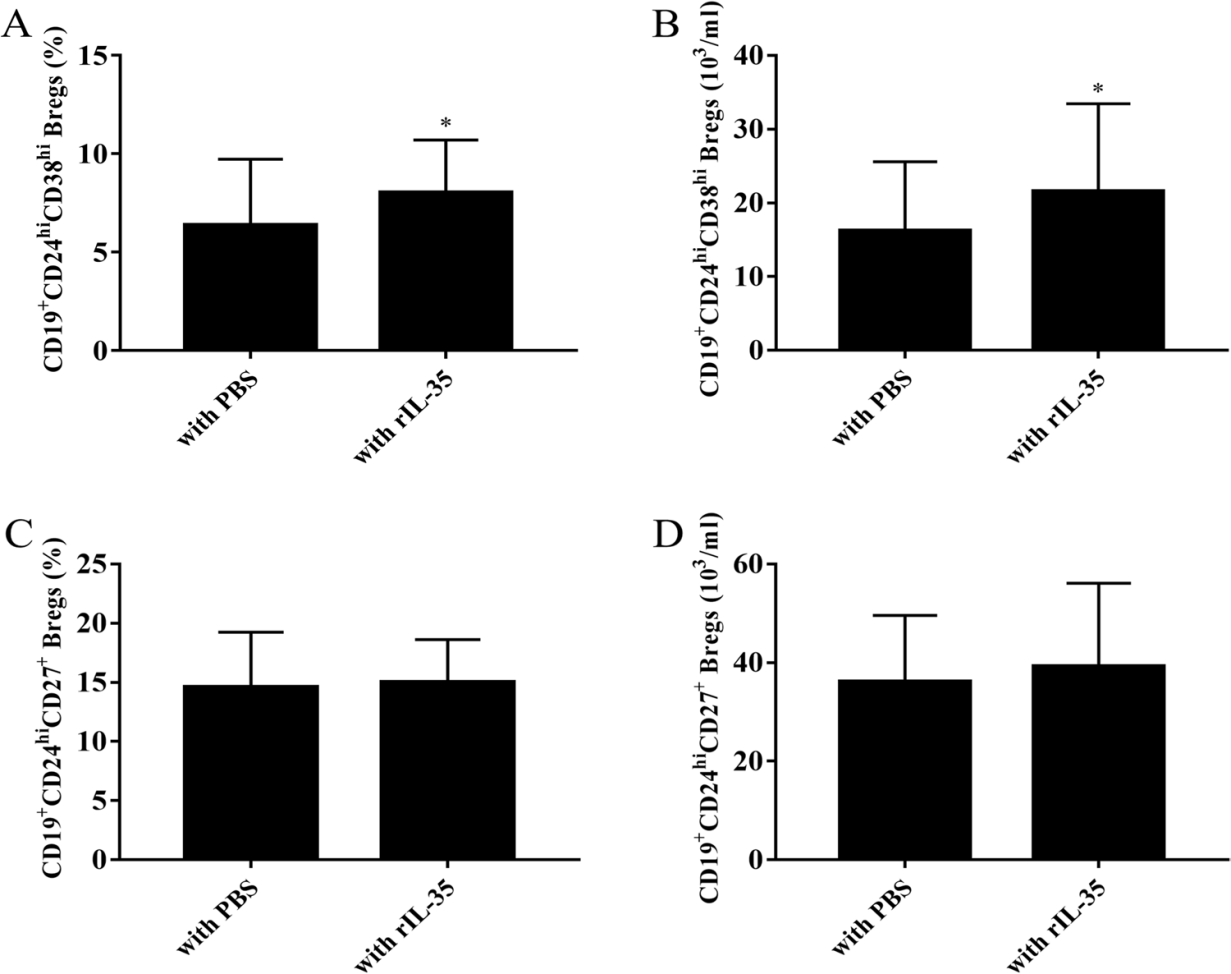
The effect of IL-35 on the expansion of Breg subsets. Freshly isolated PBMCs from AS patients (*n* = 35) were co-cultured with rIL-35 (100 ng/ml) or PBS. **A** The percentages of CD19^+^CD24^hi^CD38^hi^ Bregs was higher in cells treated with rIL-35 than in cells treated with PBS.** B **The absolute number of CD19^+^CD24^hi^CD38^hi^ Bregs was higher in cells treated with rIL-35 than in cells treated with PBS. **C **The percentages of CD19^+^CD24^hi^CD27^+^ Bregs in the rIL-35 group was not significantly different from that in the PBS group. **D** The absolute number of CD19^+^CD24^hi^CD27^+^ Bregs in the rIL-35 group was not significantly different from that in the PBS group. ^*^P < 0.05, ^**^P < 0.01 compared with the PBS group

To further determine the effect of IL-35 on Breg expansion, we measured the percentages and absolute number of CD19^+^CD24^hi^CD38^hi^ and CD19^+^CD24^hi^CD27^+^ Bregs when PBMCs from AS patients were stimulated with rIL-35 for 48 h. We observed the percentages of CD19^+^CD24^hi^CD38^hi^ Bregs in CD19^+^ B cells were significantly increased in the rIL-35-stimulated group compared to PBS-treated cells (*t* =  − 2.286, *P* = 0.026) (Fig. 7A). In addition, in the rIL-35-stimulated group also showed a increased absolute number of CD19^+^CD24^hi^CD38^hi^ Bregs (*t* = −2.081, *P* = 0.041) (Fig. 7B). Although the percentages and absolute number of CD19^+^CD24^hi^CD27^+^ Bregs was increased in the rIL-35–stimulated group, the difference with the PBS-treated group was not significant (*t* =  − 0.449, *P* = 0.655; *t*2 = −0.870, *P*2 = 0.388) (Fig. 7C and D).

### IL-35 induces Breg expansion through the STAT1 and STAT3 signaling pathways

**Fig. 8:**
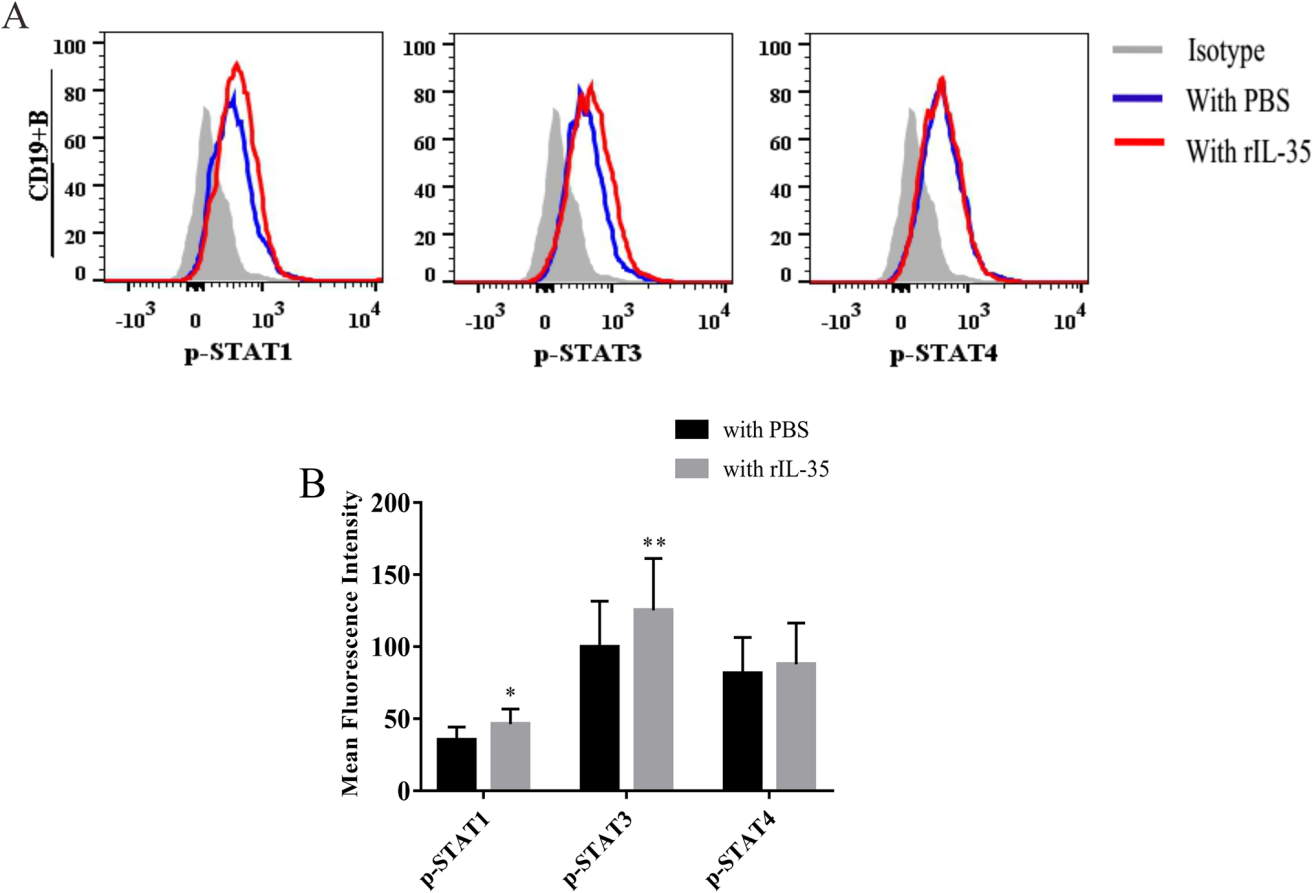
The activation of STAT signaling in CD19^+^ B cells from AS patients (*n* = 35) upon stimulation with rIL-3. **A** The phosphorylation levels of STAT1, STAT3, and STAT4 in CD19^+^ B cells with 100 ng/ml rIL-35–activated fresh isolated PBMCs were analyzed by flow cytometry. **B** The mean fluorescence intensity (MFI) of p-STAT1 and p-STAT3 were significantly increased in cells treated with rIL-35 compared with the PBS group. ^*^P < 0.05, ^**^P < 0.01 vs. the PBS group

To gain additional insight into the underlying mechanisms, we investigated the signaling pathways that IL-35 activated in Breg subsets. The STAT protein family plays a pivotal role during cellular differentiation and immunoregulation. Furthermore, STAT family transcription factors were also demonstrated to be associated with the production of IL-10 in B cells [25]. In this study, we examined the phosphorylation status of STAT1, STAT3, and STAT4 in CD19^+^ B cells of AS patients stimulated with rIL-35. As shown in Fig. 8, rIL-35 stimulation induced the phosphorylation of both STAT1 and STAT3 in CD19^+^ B cells (t =  − 4.745, *P* < 0.001; *Z* =  − 2.961, *P* = 0.003). However, the phosphorylation status of STAT4 remained unchanged (*Z* =  − 0.993, *P* = 0.321).

## Discussion

AS is a chronic inflammatory autoimmune disease that mainly affects the axial skeleton. So far, the specific causes and mechanisms of the disease have not yet been thoroughly illuminated, but immune dysfunction has been shown to be closely related to the pathogenesis of the disease. Studies have found that several subsets of immune cells, including Th1 cells, Th2 cells, Th17 cells, and Tregs, are involved in the pathogenesis of AS [26, 27]. Our previous study also showed that the percentages of follicular T helper cells and CD19^+^CD38^+^ antibody–secreting B cells were significantly increased in AS patients, which was correlated with the disease activity score [28]. Bregs form a B cell subpopulation which was recently confirmed to play an important role in the maintenance of immune homeostasis. Accumulating evidence has shown that the percentages of Bregs are aberrant in various human autoimmune diseases [9–12].

In the present study, we found that the percentages of circulating CD19^+^CD24^hi^CD38^hi^ and CD19^+^CD24^hi^CD27^+^ Bregs were decreased in AS patients compared to those in HCs, and the percentages of CD19^+^CD24^hi^CD27^+^ Bregs in patients with active AS were lower than those in patients with inactive AS, which is consistent with a previous report in psoriasis [25]. Our data also revealed that the percentages of CD19^+^CD24^hi^CD38^hi^ and CD19^+^CD24^hi^CD27^+^ Bregs are related with disease activity in patients with AS. The production of IL-10 was thought to be the primary mechanism by which Bregs exert immunosuppressive effects [5]. Simultaneously, we also demonstrated that IL-10 serum levels were significantly decreased in AS patients and that IL-10 levels are negatively associated with disease activity. Besides, the IL-10 serum levels were positively correlated with the percentages of CD19^+^CD24^hi^CD27^+^ Bregs, indicating that Bregs may serve as the main source of IL-10 in AS patients. Nevertheless, there were no significant correlations between the percentages of CD19^+^CD24^hi^CD38^hi^ Bregs and IL-10 serum levels. This result was most likely because of the low number of AS patients. Additional studies may be needed to confirm these preliminary results. The above results suggest that IL-10 production by Bregs is markedly impaired, and this reduced capacity to secrete IL-10 may go hand-in-hand with the severity of AS, demonstrating Bregs may serve a protective role in the pathogenesis of AS. Currently, the cause of the decline in Breg numbers and the impaired capacity to secrete IL-10 is unknown. Recently, studies reported that IL-35 can expand the numbers of IL-10^+^ Bregs and induce IL-10 production [29].

IL-35 has recently been considered as a new immunosuppressive and anti-inflammatory cytokine, which is composed of two subunits, i.e., p35 and EBI3. IL-35 is predominantly secreted by Tregs, has been shown to suppress Teff proliferation, induces the generation Tregs in a number of in vitro and in vivo disease models, and appears to be required for the immunosuppressive function of mouse and human Tregs [30, 31]. The immunosuppression function of IL-35 has been confirmed in multiple autoimmune diseases, including primary Sjögren’s syndrome, systemic lupus erythematosus, and HT [32–34]. In this study, we observed that the IL-35 serum levels were significantly lower in AS patients compared with HCs, and the expression levels of IL-35 were also significantly correlated with disease activity. In addition, the mRNA levels of *p35* and *EBI3* were lower in AS patients than in HCs. The lack of IL-35 may lead to low mRNA expression of *p35* and *EBI3* in AS patients. Furthermore, a positive correlation between *p35* and *EBI3* mRNA levels in HCs was observed, while there was no significant correlation between *p35* and *EBI3* mRNA levels in patients with AS. The data suggest that the two subunits of IL-35 have independent functions in regulating immunity and inflammation. Simultaneously, we also identified a positive correlation between IL-35 levels and the percentages of CD19^+^CD24^hi^CD38^hi^ and CD19^+^CD24^hi^CD27^+^ Bregs in AS patients. These findings suggested that IL-35 may be involved in the pathogenesis of AS. Combined with previous studies [16, 17], we speculated that the cause of the lower Breg counts in AS patients may be the decreased IL-35 levels, which may not be sufficient to induce the expansion of Bregs, resulting in immune dysfunction.

To test this hypothesis, we isolated PBMCs from AS patients, and then the cells were co-cultured with rIL-35 in vitro. We found that the IL-35R subunits IL-12Rβ2 and IL-27Rα were constitutively expressed on CD19^+^ B cells, and IL-12Rβ2 and gp130 were constitutively expressed on CD4^+^ T cells (data not shown) isolated from peripheral blood of AS patients, and the expression of IL-12Rβ2 and IL-27Rα on the surface of CD19^+^ B cells was significantly upregulated upon rIL-35 treatment. Moreover, IL-10 levels in the supernatant were significantly increased when PBMCs were cultured with rIL-35. This demonstrated that stimulation with rIL-35 enhanced the release of IL-10 from PBMCs. Simultaneously, we also showed that the percentages and absolute number of CD19^+^IL-10^+^ B cells and CD19^+^CD24^hi^CD38^hi^ Bregs were significantly increased when PBMCs were cultured with rIL-35. Consistent with our finding, it was recently shown that IL-35 induces conversion of B cells to Bregs that produce IL-35 as well as IL-10 [16, 17]. Furthermore, a previous study found that administration of rIL-35 attenuated collagen-induced arthritis in a mouse model [30]. The immunosuppressive properties of IL-35 in mice might be partially explained by its effect on the expansion of Tregs, the increased production of IL-10, the suppression of Teff proliferation, and the inhibition of differentiation of Th17 cells [35, 36]. The above results proved that IL-35 not only directly induced the expansion of Bregs and Tregs but also indirectly enhanced their functions by promoting the secretion of IL-10.

To better understand the effects of IL-35 on Bregs in the pathogenesis of AS, lastly, we explored the signaling pathways involved in the induction of PBMCs by rIL-35. IL-35, by binding to its receptor, recruits and activates specific members of the STAT family of transcription factors to mediate its biological effects [35, 37]. In fact, a previous study revealed that IL-35 induces the transcription of STAT1 and STAT4 in T cells and that of STAT1 and STAT3 in B cells [18, 21]. However, in contrast to a previous report [21], a study demonstrated that only STAT1 and STAT3 were activated by IL-35 expressed by tumor-infiltrating Tregs in colorectal cancer during the induction of iTr35 cells [22]. In the present study, we examined the phosphorylation status of STAT1, STAT3, and STAT4 in CD19^+^ B cells stimulated by rIL-35 by flow cytometry. The results showed that increased p-STAT1 and p-STAT3 levels in CD19^+^ B cells, and p-STAT1 and p-STAT4 levels in CD4^+^ T cells (data not shown) were observed in the rIL-35–stimulated group compared with the PBS-treated group. These data suggested the possibility that IL-35 might utilize different signaling components in different species or different types of cells. In this study, our results demonstrated that IL-35 could effectively promote the expansion of Bregs and increase IL-10 production by CD19^+^ B cells via the STAT1 and STAT3 pathways.

The limitation of our study is that it was performed in an ex vivo and in vitro setting. The in vivo effect of IL-35 on Bregs should be validated in animal models of AS. Moreover, it is unclear whether these findings shed light on a general mechanism of autoimmunity or just a phenomenon of AS.

## Conclusions

In conclusion, our results showed that IL-35 serum levels and the percentages of Breg subsets are dramatically decreased in AS and that decreased levels of IL-35 are positively correlated with the low abundance of Bregs. Meanwhile, we demonstrated that peripheral CD19^+^ B cells expressed IL-35R, and rIL-35 could effectively promote the expansion of CD19^+^CD24^hi^CD38^hi^ Bregs and increase IL-10 production by CD19^+^ B cells via the STAT1 and STAT3 pathways. These results suggest that IL-35 may play an important role in the pathogenesis of AS.

## Data Availability

No additional data available.
